# “Distracters” Do Not Always Distract: Visual Working Memory for Angry Faces is Enhanced by Incidental Emotional Words

**DOI:** 10.3389/fpsyg.2012.00437

**Published:** 2012-10-22

**Authors:** Margaret C. Jackson, David E. J. Linden, Jane E. Raymond

**Affiliations:** ^1^School of Psychology, University of AberdeenAberdeen, UK; ^2^Institute of Psychological Medicine and Clinical Neurosciences, Cardiff UniversityCardiff, UK; ^3^School of Psychology, University of BirminghamBirmingham, UK

**Keywords:** emotion, working memory, distraction, faces, facilitation, angry face, threat

## Abstract

We are often required to filter out distraction in order to focus on a primary task during which working memory (WM) is engaged. Previous research has shown that negative versus neutral distracters presented during a visual WM maintenance period significantly impair memory for neutral information. However, the contents of WM are often also emotional in nature. The question we address here is how incidental information might impact upon visual WM when both this and the memory items contain emotional information. We presented emotional versus neutral words during the maintenance interval of an emotional visual WM faces task. Participants encoded two angry or happy faces into WM, and several seconds into a 9 s maintenance period a negative, positive, or neutral word was flashed on the screen three times. A single neutral test face was presented for retrieval with a face identity that was either present or absent in the preceding study array. WM for angry face identities was significantly better when an emotional (negative or positive) versus neutral (or no) word was presented. In contrast, WM for happy face identities was not significantly affected by word valence. These findings suggest that the presence of emotion within an intervening stimulus boosts the emotional value of threat-related information maintained in visual WM and thus improves performance. In addition, we show that incidental events that are emotional in nature do not always distract from an ongoing WM task.

## Introduction

During social interaction we often have to assimilate multiple pieces of incoming information in any given moment. To achieve this we use attention systems to filter relevant from irrelevant information, and working memory (WM) to monitor, update, and integrate ongoing current events. This enables us to forecast others’ intentions and plan one’s own behavior fluently and efficiently.

Social encounters are often rich with emotion. The presence of emotion during attention and WM tasks has been shown to strongly influence how we in turn perceive and process a situation, and there is evidence that particular emotions can both facilitate and impair performance, depending on the task at hand. Numerous studies report that attention is rapidly oriented and biased toward faces displaying fear or anger (Eastwood et al., 2003; Fenske and Eastwood, 2003; Fox and Damjanovic, 2006; Hahn et al., 2006; Horstmann et al., 2006; Bannerman et al., 2010; Feldmann-Wüstefeld et al., 2011; Huang et al., 2011) and toward threatening words and scenes (Fox et al., 2001; Yiend and Mathews, 2001; Koster et al., 2004). Within the normal human population this attentional threat bias is considered to be facilitatory in that it engages a primitive survival response to locate and process danger swiftly and effectively. There is also evidence that the presence of task-irrelevant threat can enhance the allocation of attention to a separate, unrelated task. For example, contrast discrimination is shown to be more sensitive when preceded by a fearful face (Phelps et al., 2006), and visual search efficiency for a prepotent target increases when preceded by a fearful face (Becker, 2009) or an emotional scene (Kristjánsson et al., 2012). Such knock-on effects of negative emotional stimuli on attention are widely considered to result from activation of amygdala and its associated networks (e.g., Phelps et al., 2006). In contrast, negative emotions have also been shown to impair rather than facilitate attention processes. Among anxious people, for whom the threat bias is particularly pronounced (e.g., Mogg and Bradley, 1998; Bradley et al., 2000; Fox et al., 2001), attention to threatening stimuli is associated with the inability to disengage from the threat item (Fox et al., 2001, 2002; Yiend and Mathews, 2001; Koster et al., 2004), a detrimental effect which may disrupt attention to other ongoing tasks. A disadvantageous bias to threat within the normal adult population is reported in a phenomenon called emotion-induced blindness, which describes impaired awareness of stimuli that follow in close temporal and spatial proximity to a negative emotional item (Most et al., 2005). A possible mechanism for these effects is that limited attention resources for high-level visual processing are directed toward irrelevant emotional stimuli and away from task relevant ones, thus impairing performance.

A smaller number of studies have asked whether emotionally positive stimuli have effects similar to those reported for negative stimuli. The general finding is that when stimuli are associated with reward and are therefore positive, they are highly effective at attracting attention. These effects have been found with a range of different stimuli including sexual stimuli (Arnell et al., 2007; van Hoof et al., 2010), drug related stimuli for addicted individuals (e.g., Bradley et al., 2004), and for arbitrary stimuli that have been associated with reward via conditioning (Raymond and O’Brien, 2009; Anderson et al., 2011). Other work suggests that arousal level of stimuli, positive or negative, determines their effect on attention (Anderson, 2005; Schimmack, 2005). Higher-level cognitive tasks are also affected by emotion, and again both facilitatory and detrimental effects of threat are reported. Complementing the advantageous threat bias in the attention literature, visual WM for the identity of faces is significantly better for faces bearing an angry (Jackson et al., 2009) or fearful (Sessa et al., 2010) expression than for faces bearing a positive or neutral expression. The ability to accurately maintain the identity of angry and fearful individuals in visual WM is likely to have evolved from the need to respond rapidly and appropriately to social threat cues. Note that the angry and happy faces used in our previous studies (and also used here) were rated very similar in arousal levels (see Jackson et al., 2009), therefore differences in arousal are unlikely to account for the finding of enhanced WM for angry faces.

Conversely, there are also disadvantageous effects of attention to negatively valenced information on separate, concurrent WM tasks. Task-irrelevant negative stimuli that are presented during a WM task have been shown to impair memory for neutral items, termed a distraction effect. For example, negative distracter words presented during the serial presentation of word memoranda impaired serial recall relative to positive and neutral words (Buchner et al., 2004). Similarly, Dolcos and McCarthy (2006) found that visual WM for neutral faces was significantly impaired when negative versus neutral or scrambled scenes were inserted as distracters into the WM maintenance period (see Dolcos et al., 2011, for a review of the neural correlates of such emotion-cognition interactions). These findings suggest that negative information although incidental detracts attention from an ongoing neutral task in which WM is engaged [see also the review by Cohen and Henik (2012) which outlines evidence that irrelevant emotional stimuli can both impair and enhance executive control].

What is unknown at present, however, is how task-irrelevant emotional information might impact upon a separate WM task when the memoranda themselves are emotional. Effective social engagement relies heavily on WM processes to maintain and update relevant person information, and this rarely occurs in an emotional vacuum. Facial expressions of emotion are crucial for rapidly communicating one’s own state of mind, and the ability to monitor others’ emotions over time is fundamental for normal human social interaction. With this in mind, in the current study we measured visual WM for angry and happy faces and assessed the impact of incidental negative, positive, and neutral words presented during a 9 s maintenance interval. We chose to use verbal rather than visual stimuli to act as intervening distracters because this study is a precursor to an fMRI investigation. This is the first use of emotional distracters in a WM task that also involves emotional items, therefore in using fMRI we want to be able to more clearly separate and examine brain activity in regions associated with the emotional faces (visual) compared to the emotional words (verbal).

Importantly, in the WM task two angry or happy faces were presented for encoding but the single test face presented at retrieval was always neutral. The task was to state whether the test person was present or absent at encoding and emotional expression was task-irrelevant. This design ensured that any effects of word valence on WM for emotional faces could be directly attributed to the presence of facial expression information at encoding and thus maintained during the retention interval, rather than a feed-forward effect of word valence on retrieval processes if facial expression were also present at retrieval (as was the case in the original studies that first reported the angry face benefit in WM; Jackson et al., 2008, 2009).

First, we assessed whether intervening words intended to distract from the WM task would interfere with memory for emotional faces at all. Dolcos and McCarthy (2006) found that neutral distracters impaired WM for neutral stimuli relative to a condition in which a scrambled distracter was presented, indicating that incidental non-emotional information, when meaningful, can interfere with WM maintenance processes. It is possible that emotional information held in WM is protected from such distraction in a way that neutral information is not, perhaps as a result of increased salience or enhanced motivational value. To test this, in Experiment 1 we directly compared the effect of intervening neutral words versus no words on WM for angry and happy faces. We favored a no-distraction condition over a scrambled word condition in order to provide a pure baseline measure of WM performance using a maintenance interval here (9000 ms) that is nine times longer than that used in the original studies of WM for emotional faces (1000 ms; Jackson et al., 2008, 2009). We used a particularly long delay interval because in the intended follow-up fMRI experiment we aim to measure brain activity during WM maintenance. Furthermore, comparing some versus no-distraction enhances the real-life validity of the test. To anticipate, we found no difference in WM performance between the two conditions for both angry and happy faces. On the one hand, it is possible that the absence of a distracter effect resulted from the relatively long maintenance interval and/or the neutral words used in the present study. On the other hand, this result also raises the interesting possibility that, when the contents of WM are emotional, some form of protection from distraction is afforded.

In Experiment 2, we again measured WM for angry and happy faces but this time directly compared the influence of negative, positive, and neutral intervening words. With this design we can predict several possible outcomes. If attention is biased to negative words, we would expect impaired WM for both angry and happy faces when negative versus both positive and neutral words are presented during WM maintenance, an effect that should result in a main effect of word valence. There is also reason to predict an interaction between word valence and facial expression on WM performance. Negative words might impair WM for happy faces to a greater degree than WM for angry faces (and vice versa: positive words might impair WM for angry faces to a greater degree that WM for happy faces) by virtue of incompatible valence interference. Alternatively, because WM has been found to be superior for angry versus happy faces (Jackson et al., 2008, 2009), suggesting enhanced maintenance of threat information, WM for angry faces in the current task might be protected from any distraction and remain unaffected by the valence of intervening words, while WM for happy faces might suffer from negative distraction. To anticipate, none of the above predictions were borne out, making our results both surprising and interesting. We found that emotional words (regardless of whether negative or positive) actually boosted WM for angry faces relative to the neutral word condition, whereas WM for happy faces was not significantly affected by word valence. The current study adds some interesting substance to the literature concerning the ability of emotion to enhance or impair cognition. Our findings call attention to the fact that information intended to distract from an ongoing task does not always serve to impair performance; incidental emotional material can facilitate WM when the memoranda signal threat.

## Materials and Methods

### Participants

Students from Bangor University took part in return for tokens for use of university printers or money. All had normal or corrected-to-normal vision and were fluent in English. None were dyslexic (self-report). Twenty-five (20 females, 5 males; mean age 20 years) took part in Experiment 1; a different 27 (18 females, 9 males; mean age 20 years) participated in Experiment 2.

### Stimuli

We used a set of 18 male Ekman and Friesen (1976) face images, comprising six individuals each with an angry, happy, and neutral expression. Faces were grayscale with hair removed by cropping each face into an oval that when presented in the experiments at a viewing distance of 60 cm subtended approximately 3.3 × 1.9° of visual angles. It is preferable to use a small number of faces for a WM task, rather than a large stimulus set, in order to ensure that long-term memory does not encroach on any retrieval decisions. For example, if a large selection of faces were used, every time a new individual is presented at retrieval on non-match trials, participants could notice that this face had not been seen before and thus make a correct non-match judgment that is based on long-term memory rather than WM. We also chose to use only male faces for this reason, and because of the possibility that a mix of male and female faces could elicit differential responses to the emotions portrayed (a separate study is required to determine whether enhanced WM for angry faces is gender-specific).

Twenty-four words (eight negative, eight positive, eight neutral) were selected from the Affective Norms for English Words (ANEW; Bradley and Lang, 1999) database for use as distracter items presented during the WM maintenance period. Experiment 2 used all negative, positive, and neutral words while Experiment 1 used only the neutral words. Research has shown that the attention-grabbing quality of distracter words is particularly strong if the words are characterized as “other-relevant” (Wentura et al., 2000), which in turn can have social consequences for the observer/participant (Peeters, 1983). Therefore, the words used here were explicitly selected to denote person-related traits in order to strengthen the relationship between distracter items and the faces held in memory, and thus maximize the potential for distraction effects (see Table 1). Using the data provided by ANEW, the three negative, positive, and neutral word lists were matched for average word frequency and length (independent *t*-tests revealed all comparison *p*s > 0.23); positive and negative word lists were matched for arousal ratings [*t*(14) = 0.02, *p* = 0.98] but differed significantly on valence [*t*(14) = 13.94, *p* < 0.001]; the neutral list differed significantly in valence from both the negative [*t*(14) = 3.82, *p* = 0.002] and positive [*t*(14) = 18.54, *p* < 0.001] lists, and in arousal from both negative [*t*(14) = 5.58, *p* < 0.001] and positive [*t*(14) = 4.83, *p* < 0.001] lists. Words were presented in bold capital letters using Courier New font size 18. Stimuli were presented on a 22-inch Mitsubishi Diamond-Pro 2060u monitor (32-bit true color; resolution 1280 × 1024 pixels) using E-Prime 1.1 (Schneider et al., 2002).

**Table 1:** **Negative, positive, and neutral words used as distracter items, selected from the ANEW database**.

Negative	Positive	Neutral
Aggressive	Elated	Coarse
Brutal	Friendly	Detached
Cruel	Handsome	Indifferent
Evil	Honest	Listless
Hostile	Joyful	Skeptical
Ugly	Romantic	Serious
Violent	Sexy	Solemn
Wicked	Thoughtful	Weary
**MEANS**		
Valence = 2.94 (0.34)	Valence = 7.97 (0.12)	Valence = 4.36 (0.15)
Arousal = 6.16 (0.18)	Arousal = 6.16 (0.32)	Arousal = 3.63 (0.42)
Frequency = 24.13 (7.40)	Frequency = 24.63 (8.28)	Frequency = 23.25 (13.35)
Length = 6.13 (0.69)	Length = 6.88 (0.64)	Length = 7.38 (0.68)

*ANEW provides ratings of valence, arousal, frequency, and length for each word, and the means (standard errors in brackets) for each word valence category are reported here. All words were used in Experiment 2; only neutral words were used in Experiment 1*.

### Procedure and design

**Figure 1:**
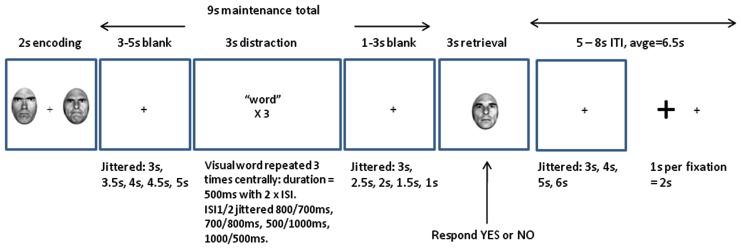
**Example trial in the “distraction” conditions**. Two angry or two happy faces were presented for encoding for 2000 ms, and 3000–5000 ms into the WM maintenance period a “distracter” word was flashed three times for 500 ms each time. Following a further 1000–3000 ms blank interval, a single neutral face was presented for retrieval. The task was to state whether the single neutral face shared identity with one of the faces present at encoding.

In summary, each trial comprised a 2000 ms WM encoding phase, a 9000 ms WM maintenance phase, and a 3000 ms WM retrieval phase. On no-distracter trials (Experiment 1 only) participants simply viewed a central fixation cross during the 9000 ms maintenance phase. On trials in which a word was presented, within the maintenance phase there was a 3000 ms “distraction” period. A trial example is provided in Figure 1. The beginning of each trial was indicated by a fixation cross that temporarily grew in size. Two faces (both angry or both happy) were then presented on either side of fixation for 2000 ms for encoding into WM. The horizontal distance between the center of each face was 3.5 cm (approximately 3.3° of visual angle). The WM maintenance period began when the faces disappeared. For the first 3000–5000 ms of the maintenance period participants simply viewed the fixation cross in the center of the screen (this variable duration was selected at random to be 3000, 3500, 4000, 4500, or 5000 ms). Then a word was flashed in the center a total of three times; each word flash was visible on the screen for 500 ms, totaling a presentation duration of 1500 ms. There was a variable delay between the first and second and between the second and third word presentations (each delay was selected to be 500, 700, 800, or 1000 ms, with the sum of the two delays totaling 1500 ms each time). This variable word gap delay was designed to reduce predictability of distracter onset and thus maximize attention to the word. Participants were instructed to simply look at the words presented. A further 1000–3000 ms variable delay period comprising only the central fixation cross (selected from 1000, 1500, 2000, 2500, or 3000 ms to always sum with the first delay period to 6000 ms) completed the 9000 ms WM maintenance interval. Finally, a single probe face that held a neutral expression was presented for retrieval from WM for 3000 ms. Participants responded “yes” if they thought the identity of the probe face matched one of the faces held in WM, and “no” if it did not match. Emotional expression was irrelevant to the task with participants required only to retain person identity information in WM. Each trial was separated by an interval comprising a single fixation cross, that varied in duration between 5000 and 8000 ms (selected at random to be 5000, 6000, 7000, or 8000 ms).

In Experiments 1 and 2, half the trials comprised angry faces and the other half happy faces. Within each face emotion condition there were equal numbers of distracter conditions (neutral versus no-distracter trials in Experiment 1; negative versus positive versus neutral trials in Experiment 2). In Experiment 1, face emotion (angry, happy) and distracter (neutral, no-distracter) conditions were pseudo-randomized with 16 trials in each condition, yielding 64 trials in total. The presentation of each face identity and word was randomized, but these factors were not fully counterbalanced with face emotion and word valence conditions as this would render the experiment too long. Each neutral word was presented four times within each condition to yield a total of 16 repetitions per word. In Experiment 2, face emotion (angry, happy) and word valence (negative, positive, neutral) conditions were also pseudo-randomized with 16 trials in each, yielding 96 trials in total. Each word was presented 4 times within each condition to yield a total of 24 repetitions per word. In both experiments, on half of trials the probe face at retrieval matched in identity to one of the faces at encoding, and on the other half it did not match, counterbalanced across face emotion and distracter conditions.

On completion of the WM task, participants rated each of the distracter words for valence and arousal using the Self Assessment Manikin (Bradley and Lang, 1994). They also rated how distracting each word seemed using a four point scale (1 = Not distracting at all; 2 = Just a little distracting; 3 = Fairly distracting; 4 = Very distracting). In support of the valence ratings provided by the ANEW database, the current sample of participants in Experiment 2 rated the negative words (mean valence = −2.59) as significantly more unpleasant than the positive words (mean valence = 2.76; *t*(26) = 18.46, *p* < 0.001) and the neutral words (mean = −0.91; *t*(26) = 8.69, *p* < 0.001). The positive words were rated as significantly more pleasant than the neutral words [*t*(26) = 19.45, *p* < 0.001]. In support of the arousal ratings provided by the ANEW database, the current sample of participants in Experiment 2 rated the negative words (mean arousal = 0.03) as similarly arousing as the positive words (mean arousal = 0.30; *t*(26) = 0.57, *p* = 0.57); the negative words as significantly more arousing than the neutral words (mean arousal = −1.68; *t*(26) = 5.76, *p* < 0.001); and the positive words as significantly more arousing than the neutral words [*t*(26) = 5.70, *p* < 0.001]. In terms of distractibility, participants from Experiment 2 rated the negative words (mean = 2.81) as significantly more distracting than the positive words (mean = 1.97; *t*(26) = 5.56, *p* > 0.001), and the neutral words (mean = 1.85; *t*(26) = 6.19, *p* < 0.001). There was a non-significant difference in distracter ratings between the positive and neutral words [*t*(26) = 1.0, *p* = 0.33].

### Data analysis

Hit rates (the proportion of trials on which participants correctly responded “yes” when the probe face matched one of the faces presented at encoding) and False Alarm rates (the proportion of trials on which participants incorrectly responded “yes” when the probe face did not match one of the faces presented at encoding) were converted into *d*′ values (*d*′ = zHits − zFalse Alarms) and submitted to statistical analysis. Reaction times (RTs), filtered to exclude responses less than than 200 ms, were analyzed on correct trials only.

## Experiment 1

In Experiment 1 we aimed to determine whether the presence of an intervening word had a distracting effect on WM for emotional faces relative to when no-distracter was present.

### Results

**Figure 2:**
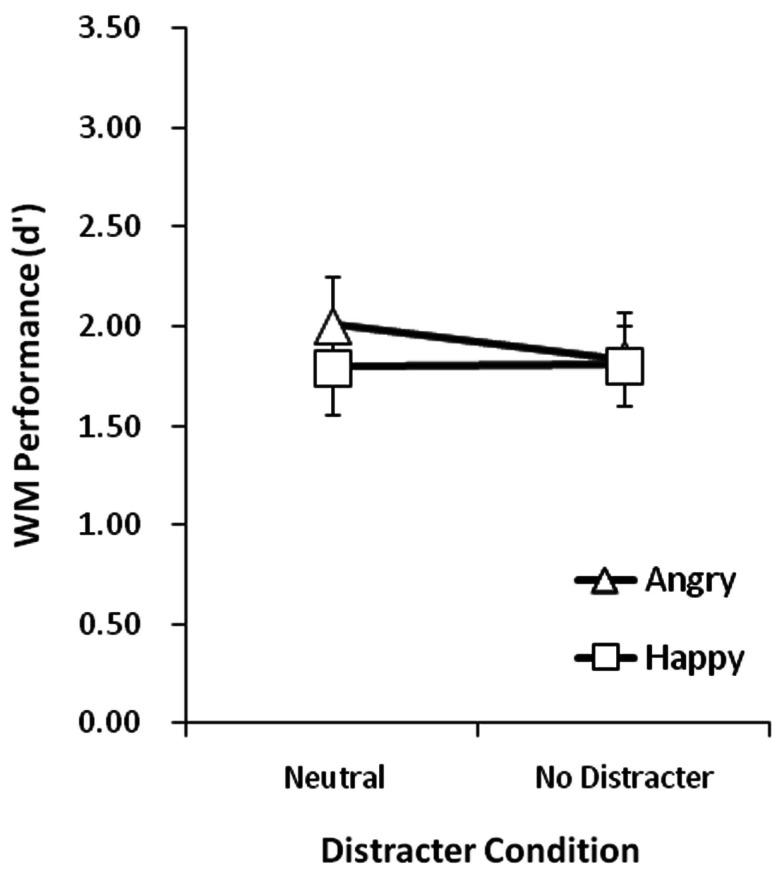
**Group mean WM performance measured in *d*′ when all WM memoranda faces had happy or angry expressions and either neutral words or no-distracter were presented during the retention interval**. Vertical lines represent ±1 SE.

A repeated-measures ANOVA on *d*′ using face emotion (angry, happy) and distracter condition (neutral, none) as within-subject factors was conducted. Group means for each condition are plotted in Figure 2. Group mean proportion correct scores are reported in Table 2. There were non-significant main effects of face emotion and distracter condition, and these two factors did not significantly interact (all *F*′s < 1.0). These results indicate that a neutral word presented during the maintenance period had no measurable impact on WM for face identity, regardless of whether the faces in memory had angry or happy expressions, relative to when no word was present. Furthermore, it is interesting to note that enhanced WM for angry versus happy faces observed in the original studies using a 1000 ms maintenance period was not replicated here (no-distracter condition) using a 9000 ms retention period.

**Table 2:** **Group mean proportion correct scores for each experiment**.

WM Condition	Distracter	Experiment 1	Experiment 2
WM Angry	Emotional		0.85 (0.02)
	Neutral	0.77 (0.02)	0.80 (0.02)
	None	0.76 (0.03)	
WM Happy	Emotional		0.81 (0.02)
	Neutral	0.76 (0.03)	0.82 (0.03)
	None	0.77 (0.02)	

*Numbers in parentheses indicate the standard error*.

A repeated-measures ANOVA on RTs, with face emotion (angry, happy) and distracter condition (neutral, none) as within factors, revealed a significant main effect of distracter condition, *F*(1, 24) = 9.53, *p* = 0.005. Responses were faster when a neutral word (mean = 1274.90 ms, SE = 46.34 ms) was presented compared to when no stimuli intervened during the retention interval (mean = 1340.34 ms, SE = 54.34 ms). It is possible that the appearance of a neutral word served to help sustain attention to the task and participants were thus perhaps better prepared to make a retrieval response, whereas when nothing happened during the long retention interval participants’ attention may have drifted and thus responses were slowed when the probe face appeared. The main effect of face emotion and its interaction with distracter condition were non-significant (both *F*s < 1.0).

## Experiment 2

In Experiment 2 we aimed to determine whether WM for angry and happy faces was differentially affected by the valence of intervening words (negative, positive, neutral) presented during maintenance.

### Results

**Figure 3:**
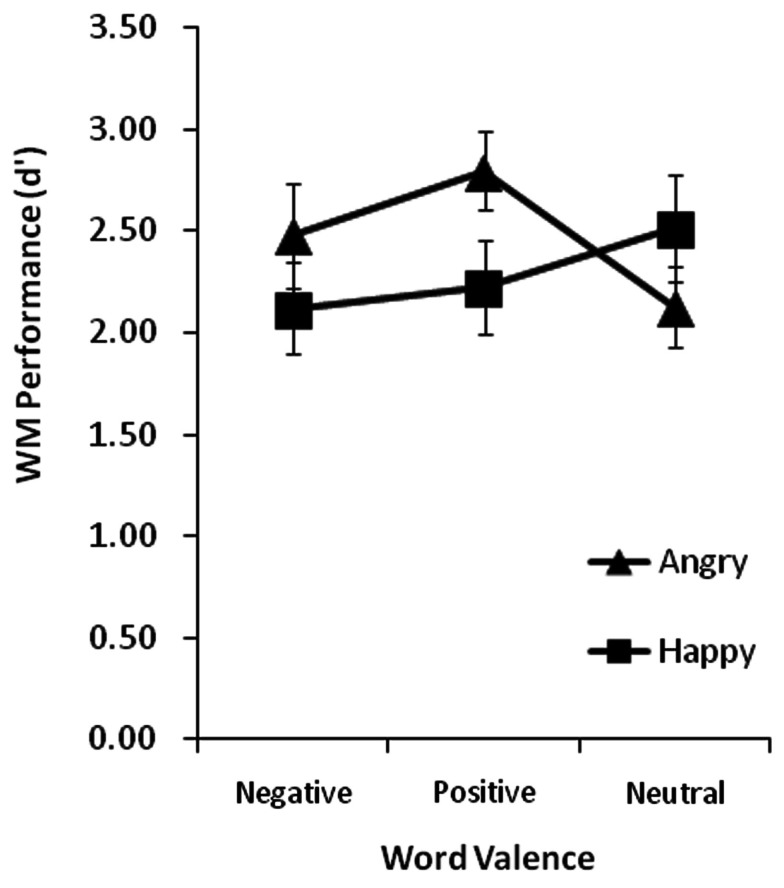
**Group mean WM performance measured in *d*′ when all WM memoranda faces had happy or angry expressions and the incidental words presented during the retention interval were negative, positive, or neutral**. Vertical lines represent ±1 SE.

A repeated-measures ANOVA on *d*′ using face emotion (angry, happy) and word valence (negative, positive, neutral) as within factors was conducted to determine distracter effects on WM for faces. Group means for each condition are plotted in Figure 3 and group mean proportion correct scores are reported in Table 2. There were non-significant main effects of face emotion [*F*(1, 26) = 1.23, *p* = 0.28] and word valence (*F* < 1.0). However, the interaction between face emotion and word valence was significant [*F*(2, 52) = 4.18, *p* = 0.02]. To explore this interaction we first examined the effects of word valence for each face emotion separately. When faces held in WM were angry, there was a significant main effect of word valence [*F*(2, 52) = 4.57, *p* = 0.02]. Paired *t*-tests revealed that WM for angry faces was significantly better when a positive (*d*′ = 2.89) versus neutral (*d*′ = 2.30) intervening word was presented, *t*(26) = 3.28, *p* = 0.003. Although WM was also better when a negative (*d*′ = 2.71) versus neutral word was presented, this difference did not reach significance, *t*(26) = 1.57, *p* = 0.13. The difference between positive and negative word conditions was non-significant, *t*(26) = 1.35, *p* = 0.19. When faces held in WM were happy, the main effect of word valence was non-significant, *F*(2, 52) = 1.14, *p* = 0.33. We also examined the presence or absence of the angry face benefit in each word valence condition. Paired *t-*tests showed that WM was significantly better for angry than happy faces when the intervening word was positive, *t*(26) = 2.44, *p* = 0.02, but the corresponding difference was non-significant when the word was negative [*t*(26) = 1.41, *p* = 0.17] or neutral [*t*(26) = 1.36, *p* = 0.19].

**Figure 4:**
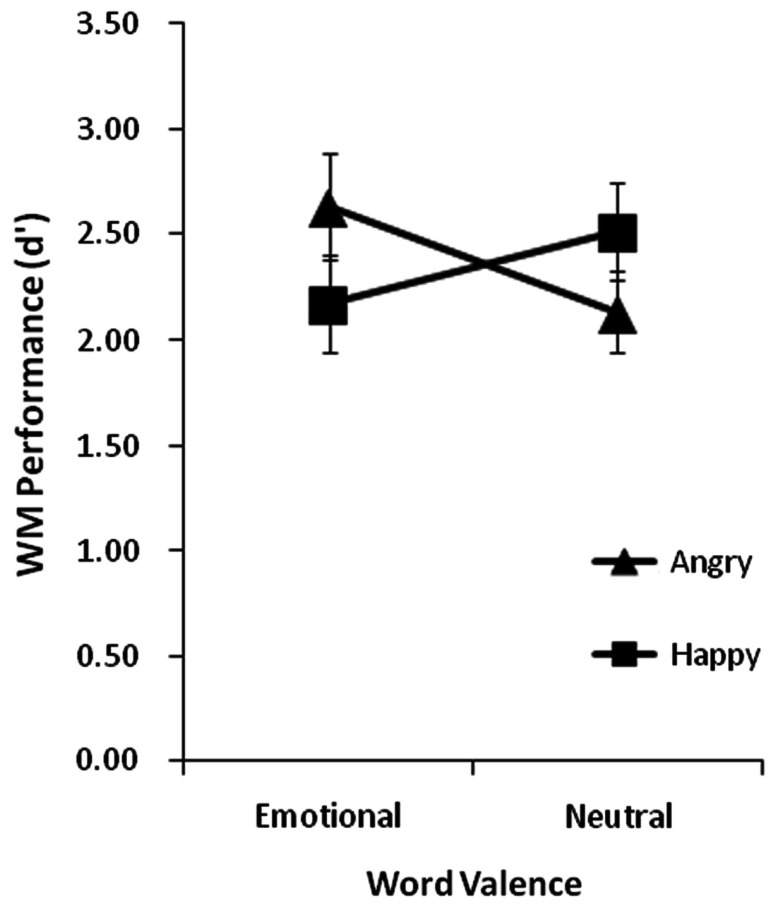
**Group mean WM performance measured in *d*′ when all WM memoranda faces had happy or angry expressions and the incidental words presented during the retention interval were emotional or neutral**. Vertical lines represent ±1 SE.

Because there was no measurable effect of positive versus negative distracters on the recall of angry faces, and also no effect on the recall of happy faces, we combined data from these two word valence conditions to compare WM when the distracter was emotional versus neutral. This data is plotted in Figure 4. A repeated-measures ANOVA with face emotion (angry, happy) and word valence (emotional, neutral) as within factors revealed a significant interaction, *F*(1, 26) = 9.14, *p* = 0.006. Main effects of face emotion and word valence were non-significant (both *F*s < 1). This interaction reflects significantly better WM for angry faces when the word was emotional (*d*′ = 2.68) versus neutral (*d*′ = 2.06) [*t*(26) = 2.85, *p* = 0.01] and a non-significant effect of word emotionality on WM for happy faces [neutral *d*′ = 2.50; emotional *d*′ = 2.13, *t*(26) = 1.56, *p* = 0.13]. Furthermore, WM was significantly better for angry than happy faces when the word was emotional [angry benefit; *t*(26) = 2.89, *p* = 0.01], but the difference in WM between angry and happy faces when the word was neutral was non-significant, *t*(26) = 1.36, *p* = 0.19.

The lack of a significant difference between WM for angry and happy faces when a neutral word was presented replicates the results of Experiment 1, and a repeated-measures ANOVA on neutral word data with face emotion as a within factor and experiment as a between factor confirmed this [non-significant face emotion × experiment interaction, *F*(1, 50) = 2.01, *p* = 0.16]. This lack of interaction also indicates that the two different participant groups performed at a similar level on the WM task in general.

Importantly, the angry face benefit in WM that we observe in Experiment 2 when an emotional word was presented during maintenance is not evident when there is no intervening word (Experiment 1). This result is particularly enlightening to the effects seen in this second experiment, as it indicates that an emotional word serves to specifically boost WM for angry faces (rather than a neutral word impairing WM), relative to the no-distracter baseline condition. WM for happy faces is less susceptible to modulation by the presence or absence of a concurrent emotional or non-emotional event. In support of these observations, independent samples *t*-tests comparing Experiments 1 and 2 revealed that when angry faces were held in WM, performance was significantly better when an emotional versus no word was presented, *t*(50) = 2.69, *p* = 0.01. When happy faces were held in memory the difference between emotional versus no word conditions was non-significant, *t*(50) = 1.43, *p* = 0.16.

A repeated-measures ANOVA on RTs, with face emotion (angry, happy) and distracter condition (negative, positive, neutral) as within factors, revealed non-significant main effects and interaction (all *F*s < 1.0).

## Discussion

The current study reveals some interesting and perhaps unexpected effects of intervening emotional stimuli on WM for emotional faces. In contrast to previous research that showed impaired WM for neutral items when a negative versus neutral distracter is presented during maintenance (Dolcos and McCarthy, 2006), here we show that emotional versus neutral words presented during the maintenance period can boost WM performance, but only when items held in WM are negative in valence (angry faces). When items held in WM are positive (happy faces), there is little evidence that the emotionality of intervening words impacts on memory performance. However, a direct comparison of our findings with those of Dolcos and McCarthy (2006) is limited because we did not include a condition with neutral WM memoranda (see Caveats section at the end).

We also found that WM for expressive (angry or happy) face identities was not significantly affected by the presentation of neutral words during the retention interval relative to when the retention interval was devoid of new stimulation, a finding that raises the possibility that emotional content in WM may afford some form of protection from distraction. Future studies can verify this possibility by confirming that distracters impair WM for neutral faces, even when using a long maintenance interval and word distracters as in the present paradigm. Alternatively, it is possible that increased task difficulty afforded by the long retention interval served to modulate the distractibility of the words. Other work in this special issue shows that task difficulty can modulate the impact of emotional distracters (Jasinska et al., 2012).

There are two possible explanations for these effects. One possibility is that when angry faces are represented in WM, their emotional significance and valence leads to a state of heightened vigilance (over and above that afforded by happy face representations) for other potentially significant emotional events, a notion consistent with the theories regarding attention biases to threat stimuli (Öhman et al., 2001). Heightened vigilance could facilitate the ability of the emotional words to compete for selection to awareness, allowing them to elaborate the face representations already in WM and thereby improve performance in the task. Although WM for angry faces did not differ significantly between positive and negative word conditions, the elevation of WM for angry compared to happy faces was driven more by positive than negative words (Figure 3). If angry faces were already deemed threatening by virtue of their expression, then negative emotional words appearing during the retention interval would be less surprising, whereas positive words would present a contradiction that could have sparked greater elaborative thought and therefore better consolidation. It is also possible that our results are due to differences in arousal levels elicited by positive and negative words. For example, the theory of *arousal-biased competition* (ABC) proposes that arousal enhances memory for items that successfully compete for selective attention (Mather and Sutherland, 2011). Despite the fact that positive and negative words were rated as equally arousing when presented outside of the WM task (and that the angry and happy faces used here were rated as equally arousing; see Jackson et al., 2009), an ABC account might suggest that angry face representations maintained in WM received a greater arousal boost when a positive word appeared than when a negative word was presented (if attention were heightened by the contradiction in valence between the face and word). However, we can only speculate about such possibilities because the same cannot be said for happy faces followed by a negative word, and we did not measure attention to the word stimuli. It is possible that in this task (which depends on WM for face identity) the task-irrelevant expression information is discarded when the to-be-remembered faces are happy but retained when they are angry. Binding expression and identity may be more imperative with negative than positive expressions because the former more typically signal a need for an immediate change in action plans, whereas the latter do not. In this view, retaining the identity of happy faces may be unaffected by word valence simply because there is no emotional information being held in WM with which the word stimuli can interact. Other work in our lab, in which we probed the emotional contents of face WM by asking participants to categorize the valence of congruent or incongruently valenced stimuli during the retention interval, suggests that this indeed might be the case (unpublished data).

Of relevance to this explanation is another interesting aspect of our results, namely that the angry face benefit to WM found in our previous work using short (1000 ms) retention intervals (Jackson et al., 2008, 2009) is not observed when the retention interval is long (9000 ms) and either lacks additional stimulation or involves the presentation of neutral words (Experiment 1). During a longer WM retention interval there is greater scope for both visual face representations and the strength of associated emotional information to fade, and this might explain why we do not find the angry face benefit here. When, as in Experiment 2, another emotional event occurs during the longer retention interval, the fading angry memory trace may be reactivated and thus enable improved performance. However, it is important to note that there are other procedural differences between the current experiment and our previous work. Here, WM load was not varied and the probe face was always neutral, making the task more difficult.

Our main result was a facilitatory effect of incidental information (the emotional words) on WM performance for face identity. This facilitatory effect conforms to a growing body of literature showing that incidental information is not necessarily distracting, but can boost performance on a range of different tasks, including WM tasks. Several studies have now shown such effects with neutrally valenced but arousing (e.g., novel or otherwise salient) stimuli. SanMiguel et al. (2010) found that whether an unexpected sound led to impaired or improved WM for neutral faces depended on trial duration. They suggested that the orienting response induced by the unexpected sound can help to refocus attention in states of unfocused attention (longer trials) whereas it may distract from the task at hand in states of more focused attention (shorter trials). Similar facilitatory effects of novel sounds were found for a visual discrimination task by Wetzel et al. (2012). Using emotional stimuli as incidental distracters, Sutherland and Mather (2012) showed that negatively arousing sounds boosted WM for perceptually salient stimuli. Additionally, positive and negative visual scenes inserted into the WM maintenance period of a delayed discrimination task for letters have been shown to support memory performance while neutral distracter scenes impaired performance, relative to a no-distracter condition (Erk et al., 2007). Taken together these findings provide an emerging picture of how incidental emotional information can support rather than hinder online processing of other information.

The novel and important finding in our study is that the facilitatory effect of incidental emotional distracters on WM for face identity was confined to the condition involving social threat in the WM memoranda. Although we previously reported a benefit for angry faces in WM without the use of incidental distracters and with a brief retention interval (1000 ms; Jackson et al., 2008, 2009), here, using a long retention interval, we found this effect only when emotional words were presented during maintenance. This suggests that an additional “boost” from incidental emotional information is needed to support the advantage of threat-related information in WM over longer intervals. Other studies have shown that emotional (versus neutral) information presented during WM retention enhances activity in “hot” emotion areas and decreases activity in “cold” executive areas during a WM maintenance period (Dolcos and McCarthy, 2006; Wong et al., 2012). While such enhanced recruitment of emotion processing areas has been shown to impair WM for neutral items, it may conversely support the consolidation of emotionally salient information, such as threat cues, into WM.

In conclusion, we find facilitatory effects of incidental information with emotional content specifically on the retention of threat-related information in WM. Our previous work had shown that angry faces are particularly well retained in WM (Jackson et al., 2008, 2009), but the present results suggest that further WM consolidation over longer periods of time relies on an added boost of activation from emotion networks. Functional imaging work is required to directly assess the impact of these results on brain activity within hot emotion and cold executive regions.

### Caveats

It is necessary to address particular aspects of our design that may raise questions in our readers. Our original findings (Jackson et al., 2009) were elicited using a paradigm which differed in several ways to the current design: (1) faces were emotional versus neutral at encoding, (2) the expression displayed at retrieval always matched the expression displayed at encoding (so on match trials at retrieval the exact same face image matched in both identity and expression was shown), and (3) a shorter retention interval of 1000 ms was used. We addressed this third point earlier in the paper and do not revisit it here, but it is important to expand upon the first two points. The original design has two disadvantages: (1) we cannot tell whether the impact of the presence of an angry expression upon WM for face identity was elicited at encoding or retrieval, (2) results may have simply been due to some sort of low level perceptual advantage in image matching afforded by angry faces, rather than a higher-level response to the presence of anger. To disentangle these issues and improve the design, we conducted a further study using angry versus happy faces at encoding but neutral faces at retrieval (with a 1000 ms retention interval). We replicated the original findings in that WM was significantly better for angry than happy faces, despite the absence of emotion at retrieval and the fact that participants were now forced to actively extract face identity from emotional expression in order to successfully perform the task (unpublished data).

We chose to use this “neutral probe” design in the current paper rather than display the same expression at encoding and retrieval as in the original study for two reasons. First, we wanted to isolate any effects of distracter word valence on WM for emotional faces to the presence of facial expression information at encoding, and avoid contamination from a possible feed-forward effect of word valence on emotion-related retrieval processes. Second, we intend to repeat the current study using fMRI to measure brain responses to emotional memoranda during the retention/distraction interval. If we were to use a design in which emotion was present in the facial memoranda during both encoding and retrieval, as well as in distracter words during the maintenance interval, this would complicate things greatly in terms of separating the brain’s response to emotional stimuli across time and context. However, we acknowledge that using the “neutral probe” design in the current paradigm carries a disadvantage in that it does not allow for a direct measure of the effect of distraction on WM for emotional versus neutral memoranda. If we were to use neutral faces at encoding (and thus neutral faces at retrieval) this introduces fundamental differences between emotion conditions in how faces are matched and retrieval decisions made, thus rendering impossible any direct comparison of the effects of distraction on emotional versus neutral faces. While our current design also limits the comparisons that can be made between our study and that of Dolcos and McCarthy (2006) in which neutral memoranda were used, we feel that our results provide valuable information on how the valence of intervening stimuli can impact differentially upon WM for positive versus negative stimuli.

It is also worth re-iterating here that our original, and other, studies showed that the presence of only specific facial emotional expressions alters how we remember non-emotion-related person information: WM for face identity was significantly better when the faces portrayed anger (versus happiness or a neutral expression; Jackson et al., 2008, 2009) or fear (versus neutral expression; Sessa et al., 2010). Importantly, our previous work showed that there was no significant difference between WM for happy and neutral faces, suggesting that it is not the presence of emotion *per se* that interacts with the WM task, but rather a specific response to threatening/negative emotions.

## Conflict of Interest Statement

The authors declare that the research was conducted in the absence of any commercial or financial relationships that could be construed as a potential conflict of interest.

## References

[B1] Anderson A. K. (2005). Affective influences on the attentional dynamics supporting awareness. J. Exp. Psychol. Gen. 134, 258–281 10.1037/0096-3445.134.2.258 15869349

[B2] Anderson B. A. Laurent P. A. Yantis S. (2011). Value-drive capture. Proc. Natl. Acad. Sci. U.S.A. 108, 10367–10371 10.1073/pnas.1014885108 21646524 PMC3121816

[B3] Arnell K. M. Killman K. V. Fijavz D. (2007). Blinded by emotion: target misses follow attention capture by arousing distractors in RSVP. Emotion 7, 465–477 10.1037/1528-3542.7.3.465 17683203

[B4] Bannerman R. L. Milders M. Sahraie A. (2010). Attentional bias to brief threat-related faces revealed by saccadic eye movements. Emotion 10, 733–738 10.1037/a0019354 21038958

[B5] Becker M. W. (2009). Panic search: fear produces efficient visual search for non-threatening objects. Psychol. Sci. 20, 435–437 10.1111/j.1467-9280.2009.02303.x 19309466

[B6] Bradley B. P. Field M. Mogg K. De Houwer J. (2004). Attentional and evaluative biases for smoking cues in nicotine dependence: component processes of biases in visual orienting. Behav. Pharmacol. 15, 29–36 10.1097/00008877-200409000-00112 15075624

[B7] Bradley B. P. Mogg K. Millar N. H. (2000). Covert and overt orienting of attention to emotional faces in anxiety. Cogn. Emot. 14, 789–808 10.1080/02699930050156636

[B8] Bradley M. M. Lang P. J. (1994). Measuring emotion: the self-assessment manikin and the semantic differential. J. Behav. Ther. Exp. Psychiatry 25, 49–59 10.1016/0005-7916(94)90063-9 7962581

[B9] Bradley M. M. Lang P. J. (1999). Affective Norms for English Words (ANEW): Stimuli, Instruction Manual and Affective Ratings. Technical Report C-1. Gainesville, FL: The Center for Research in Psychophysiology, University of Florida.

[B10] Buchner A. Rothermund K. Wentura D. Mehl B. (2004). Valence of distracter words increases the effects of irrelevant speech on serial recall. Mem. Cognit. 32, 722–731 10.3758/BF03195862 15552349

[B11] Cohen N. Henik A. (2012). Do irrelevant emotional stimuli impair or improve executive control? Front. Int. Neurosci. 6:33 10.3389/fnint.2012.00033 PMC3376948 22719722

[B12] Dolcos F. Iordan A. D. Dolcos S. (2011). Neural correlates of emotion-cognition interactions: a review of evidence from brain imaging investigations. J. Cogn. Psychol. 23, 669–694 10.1080/20445911.2011.594433 PMC3206704 22059115

[B13] Dolcos F. McCarthy G. (2006). Brain systems mediating cognitive interference by emotional distraction. J. Neurosci. 26, 2072–2079 10.1523/JNEUROSCI.5042-05.2006 16481440 PMC6674921

[B14] Eastwood J. D. Smilek D. Merikle P. M. (2003). Negative facial expression captures attention and disrupts performance. Percept. Psychophys. 65, 352–358 10.3758/BF03194566 12785065

[B15] Ekman P. Friesen W. V. (1976). Pictures of Facial Affect. Palo Alto, CA: Consulting Psychologists Press

[B16] Erk S. Kleczar A. Walter H. (2007). Valence-specific regulation effects in a working memory task with emotional context. Neuroimage 37, 623–632 10.1016/j.neuroimage.2007.05.006 17570686

[B17] Feldmann-Wüstefeld T. Schmidt-Daffy M. Schubö A. (2011). Neural evidence for the threat detection advantage: differential attention allocation to angry and happy faces. Psychophysiology 48, 697–707 20883506 10.1111/j.1469-8986.2010.01130.x

[B18] Fenske M. J. Eastwood J. D. (2003). Modulation of focused attention by faces expressing emotion: evidence from flanker tasks. Emotion 3, 327–343 10.1037/1528-3542.3.4.327 14674827

[B19] Fox E. Damjanovic L. (2006). The eyes are sufficient to produce a threat superiority effect. Emotion 6, 534–539 10.1037/1528-3542.6.3.534 16938095 PMC1852642

[B20] Fox E. Russo R. Bowles R. Dutton K. (2001). Do threatening stimuli draw or hold visual attention in subclinical anxiety? J. Exp. Psychol. Gen. 130, 681–700 10.1037/0096-3445.130.4.681 11757875 PMC1924776

[B21] Fox E. Russo R. Dutton K. (2002). Attentional bias for threat: evidence for delayed disengagement from emotional faces. Cogn. Emot. 16, 355–379 10.1080/02699930143000527 18273395 PMC2241753

[B22] Hahn S. Carlson C. Singer S. Gronlund S. D. (2006). Ageing and visual search: automatic and controlled attentional bias to threat faces. Acta Psychol. (Amst) 123, 312–336 10.1016/j.actpsy.2006.01.008 16524554

[B23] Horstmann G. Borgstedt K. Heumann M. (2006). Flanker effects with faces may depend on perceptual as well as emotional differences. Emotion 6, 28–39 10.1037/1528-3542.6.1.28 16637748

[B24] Huang S.-L. Chang Y.-C. Chen Y.-J. (2011). Task-irrelevant angry faces capture attention in visual search while modulated by resources. Emotion 11, 544–552 10.1037/a0022578 21517157

[B25] Jackson M. C. Wolf C. Johnston S. J. Raymond J. E. Linden D. E. J. (2008). Neural correlates of enhanced visual short-term memory for angry faces: an fMRI study. PLoS ONE 3, e3536 10.1371/journal.pone.0003536 18958158 PMC2568825

[B26] Jackson M. C. Wu C.-Y. Linden D. E. J. Raymond J. E. (2009). Enhanced visual short-term memory for angry faces. Exp. Psychol. Hum. Percept. Perform. 5, 363–374 10.1037/a0013895 19331494

[B27] Jasinska A. J. Yasuda M. Rhodes R. E. Wang C. Polk T. A. (2012). Task difficulty modulates the impact of emotional stimuli on neural response in cognitive-control regions. Front. Psychol. 3:345 10.3389/fpsyg.2012.00345 23060828 PMC3464044

[B28] Koster E. H. Crombez G. Van Damme S. Verschuere B. De Houwer J. (2004). Does imminent threat capture and hold attention? Emotion 4, 312–317 10.1037/1528-3542.4.3.312 15456400

[B29] Kristjánsson A. Óladótir B. Most S. B. (2012). “Hot” facilitation of “cool” processing: emotional distraction can enhance priming of visual search. J. Exp. Psychol. Hum. Percept. Perform. [Epub ahead of print]. 10.1037/a0028683 22642218

[B30] Mather M. Sutherland M. R. (2011). Arousal-biased competition in perception and memory. Perspect. Psychol. Sci. 6, 114–133 10.1177/1745691611400234 21660127 PMC3110019

[B31] Mogg K. Bradley B. P. (1998). A cognitive-motivational analysis of anxiety. Behav. Res. Ther. 36, 809–848 10.1016/S0005-7967(97)00062-4 9701859

[B32] Most S. B. Chun M. M. Widders D. M. Zald D. H. (2005). Attentional rubber necking: cognitive control and personality in emotion-induced blindness. Psychon. Bull. Rev. 12, 654–651 10.3758/BF03196754 16447378

[B33] Öhman A. Flykt A. Esteves F. (2001). Emotion drives attention: detecting the snake in the grass. J. Exp. Psychol. Gen. 130, 466–478 10.1037/0096-3445.130.3.466 11561921

[B34] Peeters G. (1983). “Relational and informational patterns in social cognition,” in Current Issues in European Social Psychology, eds Doise W. Moscovici S. (Cambridge: Cambridge University Press), 201–237

[B35] Phelps E. A. Ling S. Carasco M. (2006). Emotion facilitates perception and potentiates the perceptual benefits of attention. Psychol. Sci. 17, 292–299 10.1111/j.1467-9280.2006.01701.x 16623685 PMC1555625

[B36] Raymond J. E. O’Brien J. L. (2009). Selective visual attention and motivation: the consequences of value learning in an attentional blink task. Psychol. Sci. 20, 981–988 10.1111/j.1467-9280.2009.02391.x 19549080

[B37] SanMiguel I. Morgan H. M. Klein C. Linden D. E. J. Escera C. (2010). On the functional significance of Novelty-P3: facilitation by unexpected novel sounds. Biol. Psychol. 83, 143–152 10.1016/j.biopsycho.2009.11.012 19963034

[B38] Schimmack U. (2005). Attentional interference effects of emotional pictures: threat, negativity, or arousal? Emotion 5, 55–66 10.1037/1528-3542.5.1.55 15755219

[B39] Schneider W. Eschman A. Zuccolotto A. (2002). E-Prime User’s Guide. Pittsburgh: Psychology Software Tools Inc

[B40] Sessa P. Luria R. Gotler A. Jolicoeur P. Dell’Acqua R. (2010). Interhemispheric ERP asymmetries over inferior parietal cortex reveal differential visual working memory maintenance for fearful versus neutral facial identities. Psychophysiology 48, 187–197 10.1111/j.1469-8986.2010.01046.x 20557488

[B41] Sutherland M. R. Mather M. (2012). Negative arousal amplifies the effects of saliency in short-term memory. Emotion.[Epub ahead of print]. 10.1037/a0027860 22642352 PMC3586810

[B42] van Hoof J. C. Crawford H. van Vugt M. (2010). The wandering mind of men: ERP evidence for gender differences in attention bias toward attractive opposite sex faces. Soc. Cogn. Affect. Neurosci. 6, 477–485 10.1093/scan/nsq066 20601424 PMC3150857

[B43] Wentura D. Rothermund K. Bak P. (2000). Automatic vigilance: the attention-grabbing power of approach-and avoidance-related social information. J. Pers. Soc. Psychol. 78, 1024–1037 10.1037/0022-3514.78.6.1024 10870906

[B44] Wetzel N. Widmann A. Schroeger E. (2012). Distraction and facilitation – two faces of the same coin? J. Exp. Psychol. Hum. Percept. Perform. 38, 664–674 10.1037/a0025856 22022895

[B45] Wong G. Dolcos S. Denkova E. Morey R. Wang L. McCarthy G. (2012). Brain imaging investigation of the impairing effect of emotion on cognition. J. Vis. Exp. 60, pii: 2434. 10.3791/2434 PMC3369638 22330776

[B46] Yiend J. Mathews A. (2001). Anxiety and attention to threatening pictures. Q. J. Exp. Psychol. A 54, 665–681 10.1080/02724980042000462 11548029

